# Tracking the Insider Attacker: A Blockchain Traceability System for Insider Threats

**DOI:** 10.3390/s20185297

**Published:** 2020-09-16

**Authors:** Teng Hu, Bangzhou Xin, Xiaolei Liu, Ting Chen, Kangyi Ding, Xiaosong Zhang

**Affiliations:** 1 Institute for Cyber Security, School of Computer Science and Engineering, University of Electronic Science and Technology of China, Chengdu 611731, China; mailhuteng@gmail.com (T.H.); brokendragon@uestc.edu.cn (T.C.); kangyiding@gmail.com (K.D.); 2 Institute of Computer Application, China Academy of Engineering Physics, Mianyang 621900, China; xbw@mail.ustc.edu.cn (B.X.); liuxiaolei@caep.cn (X.L.); 3 School of CyberScience, University of Science and Technology of China, Hefei 230027, China

**Keywords:** blockchain, insider threat, traceability system, differential privacy

## Abstract

The insider threats have always been one of the most severe challenges to cybersecurity. It can lead to the destruction of the organisation’s internal network system and information leakage, which seriously threaten the confidentiality, integrity and availability of data. To make matters worse, since the attacker has authorized access to the internal network, they can launch the attack from the inside and erase their attack trace, which makes it challenging to track and forensics. A blockchain traceability system for insider threats is proposed in this paper to mitigate the issue. First, this paper constructs an insider threat model of the internal network from a different perspective: insider attack forensics and prevent insider attacker from escaping. Then, we analyze why it is difficult to track attackers and obtain evidence when an insider threat has occurred. After that, the blockchain traceability system is designed in terms of data structure, transaction structure, block structure, consensus algorithm, data storage algorithm, and query algorithm, while using differential privacy to protect user privacy. We deployed this blockchain traceability system and conducted experiments, and the results show that it can achieve the goal of mitigating insider threats.

## 1. Introduction

Some networks always have high-security requirements due to their specificities, such as the internal networks of governments, enterprises, scientific research, the military, and other confidential organizations. They are generally physically isolated, that is, not connected to the Internet, and only used as the internal offices to perform research, data exchange, etc. Typically, external attacks are relatively less threatening to such network, while more likely to be threatened from inside [1]. Because of the amount of sensitive information that exists in such networks, insider threats can be devastating and can cause incalculable damage and loss to governments and organizations, as such attacks are carried out by authorized and trusted insiders who have legitimate access to the system [2].

Once an insider threat has occurred, it becomes exceedingly difficult to trace and obtain evidence. The attacker is a legitimate user, and possibly administrators of the system. Thus, the security facilities of the internal network cannot monitor or block such attacks [3]. Even if there are security logs, the attacker may delete them through other means. Moreover, the perpetrator of the insider attack may be a group, which makes it more difficult to trace the source of insider threats.

Therefore, to mitigate this issue, it is necessary to ensure the integrity of user behaviour data in the internal network system. It is also necessary to achieve traceability of user behaviour and ensure tracking information cannot be tampered with. For an internal network with high-security requirements, user identity information, user behavior information, file data, etc. are all sensitive information. Hence, it is required to protect the privacy of these data while satisfying integrity and reliability.

This paper proposes a blockchain traceability system. The system uses the characteristics of immutability and decentralization of blockchain technology to ensure the data integrity [4]. This paper designs the blockchain system from the aspects of data structure, transaction structure, block structure, consensus algorithm, data storage algorithm, query algorithm, etc., while using differential privacy to protect user privacy. The main contributions of this paper are as follows:

1. This paper builds an insider threat model of the internal network based on real insider threat situations, i.e., insider attack forensics and preventing insider attackers from escaping, and analyzes why it is difficult to trace the attacker and obtain evidence after the insider threat occurred.
2. A blockchain traceability system for insider threats is designed in this paper, which can ensure the integrity of traceable data and protect the privacy of sensitive information.
3. Experiments show that the blockchain traceability system proposed in this paper is capable of tracking data while protecting user privacy, allowing these contradictory properties to coexist.

The rest of the paper is organized as follows. Section 2 briefly reviews the related work of insider threat, blockchain, and differential privacy. Section 3 introduces the threat model. In Section 4, the design of our blockchain traceability system is described in detail. Section 5 presents our experiment and discussion. Finally, Section 6 concludes the paper.

## 2. Background and Related Work

The insider threat has always been one of the most serious challenges to cybersecurity [5,6]. The attacker has legal access to the internal network system [7]. Furthermore, they possibly have a good understanding of the system’s security policies and means and thus can easily bypass the system’s security facilities [8]. The insider attack is happening when insider adversaries destroy or steal confidential organizational information for personal gain [9]. Furthermore, in some confidential organizations, the insider threat attacks may even be spy activities at the national level [10]. Therefore, insider threats can lead to the destruction of the organization’s internal network system and information leakage, which greatly threatens the confidentiality, integrity, or availability of data in the system [11]. In order to address this issue, many researches have been done on the detection of insider threats. Take some examples, Ref. [12] proposed an ontological framework and an approach for improving physical security and insider threat detection, which can use rule-based anomaly detection to facilitate forensic data analysis and proactively mitigate insider threats. Ref. [13] used machine learning algorithms and extracted common words from topic modeling techniques, and then validated the analysis results by matching them to information security compliance elements to find potentially malicious insiders from social media. Ref. [14] implemented a fuzzy classifier as well as a genetic algorithm to improve the efficiency of the fuzzy classifier and the functionality of all other modules to obtain better results in terms of false alarms. Ref. [15] applied the Cryptomarkov method to the CERT (Computer Emergency Response Team) dataset and analyzed multiple distance-vector methods to detect changes in behavior that were shown to be successful in identifying different insider threats. Ref. [1] proposed a scalable system for high-throughput real-time analysis of heterogeneous data flows, which can be well applied to detect attacks such as financial fraud, and network intrusions etc. Ref. [3] proposed an insider threat detection method based on mouse biometrics and CNN networks with good results. Ref. [16] used blockchain to enhance the effectiveness of Bayesian inference based trust management, to help improve the efficiency of detecting malicious nodes with a reasonable workload.

However, there is little research on the traceability of insider threats. One of the main reasons is after an attacker launches an attack from inside, the attacker would also erase the attack behavior, which makes it challenging to trace and obtain evidence after an insider threat. The blockchain technology has emerged in recent years can well protect the integrity of data. Thus, it can effectively prevent the trace of insider attackers from being erased. Furthermore, there has been some research in this area. Ref. [17] proposed MeDShare based on blockchain, which addresses the problem of medical data sharing among medical big data custodians in a distrustful environment. Ref. [18] proposed a safe and effective way to achieve EMR data sharing, called BPDS, where the original EMR is stored securely in the cloud, and the index is kept in the tamper-proof alliance blockchain. Ref. [6] developed a framework that integrates the Ethereum blockchain with edge computing to perform checks and preserve the integrity of incoming sensor data before it is analyzed, processed, and stored. Ref. [19] proposed a decentralized runtime monitoring architecture based on blockchain technology to improve the accountability and reliability of distributed access control systems.

Blockchain itself is a good option for enhanced security and trust, but it is not yet pre-equipped with any privacy protection technology [20]. Nonetheless, one of the main problems in the practical application of blockchain is still privacy and information leakage [20]. Therefore, before the actual implementation of blockchain, it is very necessary to integrate privacy protection technology. Although the blockchain provides pseudo-anonymity through key encryption, this pseudo-anonymity is not sufficient to provide a complete privacy guarantee [21]. This is because any anonymous data can be combined with similar data sets to reveal personal information [22]. Differential privacy is a practical and feasible solution for blockchain privacy protection, as its dynamic nature and strong theoretical guarantees [23].

Differential privacy [24] is a new definition of privacy that Dwork proposed in 2006 in response to privacy breaches in statistical databases. Differential privacy can address the shortcomings of traditional privacy protection models [25,26,27]. One of them is that the differential privacy protection model assumes that the attacker can access the information of all records except the target record. The sum of this information can be understood as the maximum background knowledge that the attacker can master. There are two main models of differential privacy: the central model and the local model [28]. In the central model, users provide their data to a trusted data administrator, who runs a complete privacy mechanism on the data set. In the local model, the user implements a privacy mechanism before sending data to the data administrator. Local differential privacy (LDP) [29] provides stronger privacy guarantees than central differential privacy because it does not rely on the assumption of a trusted data administrator. Due to the distributed nature of the blockchain network, using local differential privacy may be a more feasible solution than using centralized differential privacy [20].

## 3. Threat Model

There have been many studies that have systematically analysed insider threats, and these works have modelled them from different perspectives. (1) Some researchers build insider threat models based on insiders, such as: Ref. [10] proposes a framework of risk factor modeling for insider threats based on the four components of organization, environment, system and individual and their interactions. Ref. [30] models consecutively connected aspects of an insider incident: catalysts, actor characteristics, attack characteristics and organizational characteristics. Ref. [31] proposed an insider threat theory model that combines the concept of CMO (Capability, Motive, and Opportunity), the theory of planned behavior, and dark triad personality traits. (2) Some researchers have established insider threat models based on system dynamics. Ref. [32] describes a System Dynamics model of the insider IT sabotage problem that elaborates complex interactions in the domain and unintended consequences of organizational policies, practices, technology, and culture on insider behavior. In their follow-up work, Ref. [33] describes general observations about and a preliminary system dynamics model of this class of insider crime based on their empirical data. (3) Some works model insider threats through formalization methods. Ref. [34] uses Bayesian network and Markov decision processes formally model the malicious insider threat at micro and macro levels, respectively. Ref. [35] uses attack trees and the interactive theorem prover Isabelle to formally model malicious and unintentional insider threats in IoT. (4) Some researchers model the insider threat based on psychological and sociological models. For example, Ref. [36] introduces a Bayesian network model for the motivation and psychology of the malicious insider. (5) Since the insider threat is also a confrontation between attackers and defenders in the internal network system, some researchers have introduced game theory into the insider threat model. Ref. [8] proposes a game-theoretic model for the insider problem, which they call an “insider game” between a system administrator and an insider attacker. Ref. [37] proposes two novel algorithms for reputation establishment, which can significantly improve the performance of anomaly detection against insider attacks in terms of the tradeoff between detection and false positives. Ref. [38] apply game theory to model the interactions between insiders and systems in an extensive form game. (6) Some research works focus on how to defend against and detect insider threats. Ref. [39] presents a model that can prevent malicious insider activities at the database application level. Ref. [40] presents a novel, interdisciplinary insider threat prediction model, which combines approaches, techniques, and tools from computer science and psychology. Ref. [41] proposes a model called SIDD (Sensitive Information Dissemination Detection) system which is aims to address methods to detect, deter and prevent deliberate and unintended distribution of sensitive content outside the organization using the organization’s system and network resources by a trusted insider. Ref. [42] models user search behavior and uses SVM to detect deviations indicating a masquerade attack. Ref. [43] uses PLSI to model the employees’ interests from e-mail to identifying individuals who feel alienated from the organisation or have a hidden interest in a sensitive topic.

This paper models the insider threat from a different perspective: insider attack forensics and prevents insider attacker from escaping. The insider attacker is located in the internal network environment and has legitimate access to the internal. The attacker’s traces are more capable of being erased than those of an external attacker, which makes tracing and forensics of insider threats extremely challenging. Based on this vulnerability, this paper builds a threat model and designed the blockchain traceability system to record all related actions of a malicious insider before, during, and after conducting an incident, while preventing attackers from erasing traces of their actions on internal network systems.

The threat in this paper is assumed to come from inside, and it can be summarized in two forms: the individual attack and the group attack.

Individual attack: from the aspect of user roles, this attack can be divides into administrator attack and normal user attack. The administrator adversary can erase, tamper with, forge, or destroy the true record of its activities in the internal system; while the normal adversary, may be a normal employee who can access to the internal systems to steal the information. However, it’s also possible that the normal user attacker can obtain the administrator account without being discovered by security detection facilities. For example, an attacker might be the developer of a security audit system, so analysing the audit logs might be able to obtain accounts and passwords of the system users. Or the other way around, an attacker can take advantage of the administrator’s negligence and use the administrator device to access the system.

Group attack: this attack means there are many adversaries cooperate to conduct an inside attack. Specifically, there is someone responsible for launching an attack to steal secrets information; someone is responsible for covering up the true attack intentions; after an insider threat occurs, someone with a high position uses the position to hinder the investigation and evidence collection.

**Figure 1:**
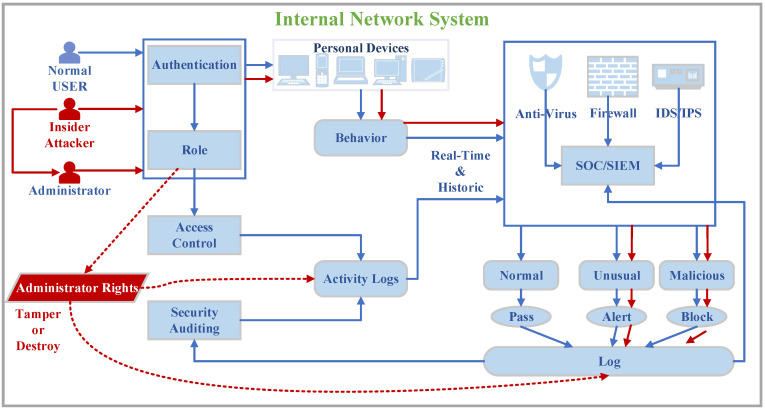
The insider threat model on the internal network system.

Figure 1 illustrates an internal network system with a relatively well secured facility. The activities of normal users in the internal network are shown by the solid blue lines in the figure. Normal users login to personal devices on the internal network through identity authentication and their permissions are determined by the access control system. All behaviors of users on the internal network are subject to access control supervision, and real-time detection of firewalls, antivirus, and IDS/IPS (short for Intrusion Detection System/Intrusion Prevention system). The activity logs, security logs, and system logs generated in the internal network are linked with SOC/SIEM (short for Security Operations Center/Security Information and Event Management) for correlation analysis to achieve unknown attack detection. There is also a security audit system to report system security trends regularly.

However, all these means may not help in the face of insider threats. The solid red lines in the figure show the activities of malicious users in the internal network, it explained how how the insider threat happen. When an insider attack occurs, the attacker pretends to be their user through their legitimate account. Then, the attacker logs into the internal network device to perform malicious activities such as copying sensitive information and destroying digital assets. Since the attacker is a legally authorized user in the internal network, these activities may not be regarded as malicious behavior by security facilities, even the IDS, SOC, security audit, etc. have discovered the unusual behavior through correlation analysis. Subsequently, the attacker would erase, tamper, or destroy the activity logs through administrator rights after the attack is completed, as shown by the red dotted line. That is why it is challenging to locate the attacker after the insider threat event, and it is also exceedingly difficult to trace and obtain evidence of the attack.

## 4. System Design

### 4.1. The Framework of the System

This paper designed a blockchain traceability system to mitigate the issue of insider threat. The system has three characteristics: nodes are divided into management nodes and participation nodes; each node maintains two blockchains, include privacy-chain and self-chain; on the premise of not affecting the traceability function, user privacy is protected by differential privacy.

First, the blockchain system is applied within the organization, and all participants are insiders. However, considering the division of authority between departments, the insiders in each department should be managed by the department itself. Therefore, the nodes are divided into management nodes and participation nodes according to different permissions. The management node is the master node of each department. It is mainly responsible for generating blocks, verifying the true traceability information sent by participation nodes, and maintaining the privacy-chain and the department’s self-chain. The participation nodes are ordinary users in the internal network system, which are responsible for packaging the traceability data generated by the users into transactions and sending them to the management node of their department, and maintaining the privacy-chain and their own self-chain. Each department has a management node and many participation nodes.

The second is that each node in the system maintains two blockchains. One is the main chain in which all nodes participate in synchronization, which is the so called privacy-chain. The traceability information on the privacy-chain only retains the department name, username, timestamp, and other information which not affecting the traceability function. The user’s key privacy information, such as their activities on the internal network, is protected by differential privacy. This chain guarantees the integrity and traceability of the data. The other is that each node maintains its own chain, which is a copy of the privacy chain. However, each node retains its own true traceability information, called self-chain, which has not been processed by differential privacy. Moreover, the self-chain in the participation node only contains its own true traceability information, but the self-chain in the management node contains all the true traceability information of all participating nodes under the jurisdiction of this department.

The third is the differential privacy protection of node data. Differential privacy is a rigorous mathematical framework that formally defines the privacy properties of data analysis algorithms [44]. Informally it requires that any changes to a single data point in the dataset can only cause statistically insignificant changes to the output of the query operation. This technology not only protects the privacy of users, but also makes the statistical properties of the entire dataset not to be destroyed, so that it can be used for secondary development or use by third parties. This paper uses a random response mechanism to protect privacy-related data, and control the degree of data protection through privacy parameter ε [45]. In this paper, the random response mechanism can be regarded as a filter. When outputting discrete data containing privacy information, first send the data to a random response mechanism, which randomly selects the output value among the candidate values according to the rules to achieve the protection of data privacy.

**Figure 2:**
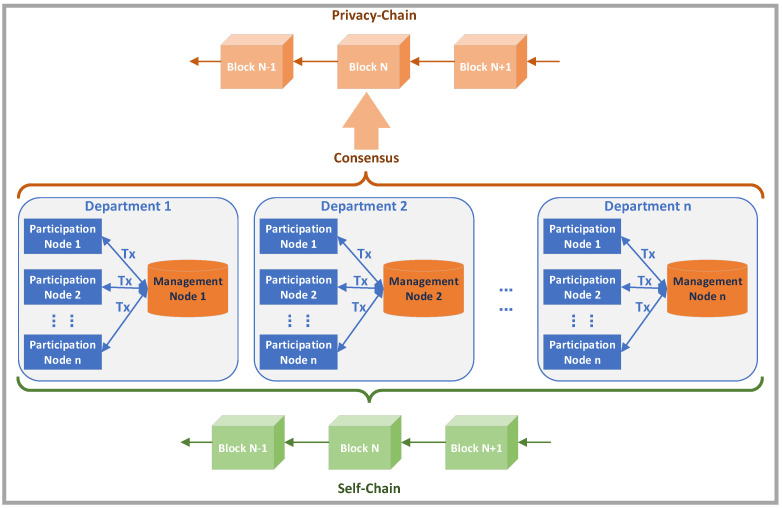
Schematic diagram of the system framework.

The schematic diagram of the system framework is shown in Figure 2. Section 4.2 describes the design of the data structure. Section 4.3 describes the design of the structure of transaction and block. Section 4.4 describes the design of differential privacy. The consensus algorithm, traceability data storage and query algorithm are described in Section 4.5 and Section 4.6.

### 4.2. Data Structure

**Figure 3:**
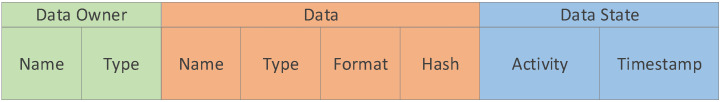
The traceability data structure.

In response to insider threats, this paper has carried out a detailed data structure design on the traceability object. The traceability object can be a user, a file, or other data types in the internal network system. In this paper, only use the types of “user” and “file” to illustrate the data structure, but other types of data can be supported by extending objects in practical applications. The traceability data structure is shown in Figure 3.

The data structure has three parts:

- Data Owner: Indicates who generated the data in the first place or who is operating on the data, including the name and type fields.
- Data: Represents the information of the data itself, including four fields of name, type, format, and hash value of the data.
- Data State: indicates the state of the data at the time, including activity and timestamp fields.

According to the defined data structure, the traceability data (hereafter referred to as TD) can be expressed as:

TD = [DataOwner, Data, DataState]

The DataOwner contains the name and type of the data owner, i.e.,:

DataOwner = [DataOwnerName, DataOwnerTpye]

where, DataOwnerName is the name of the data owner, and DataOwnerTpye refers to the type of the data owner.

Data fields include: DataName, the name of the data itself; DataType, the type of the data; DataFormat, the format of the data; DataHash, the hash value of the data itself, i.e.,

Data = [DataName, DataType, DataFormat, DataHash]

Furthermore, in the DataState, Activity represents for the state or the behavior of the data, such as user login, user open file, file creation, etc. The timestamp is the time when the Activity happened, i.e.,

DataState = [Activity, Timestamp]

Therefore, the TD structure can be expressed as:

TD = [[DataOwnerName, DataOwnerTpye], [DataName, DataType, DataFormat, DataHash], [Activity, Timestamp]]

Here is an example to illustrate the specific use of TD. User A of department 1 logs into device 1 in the intranet at time t, then the structure can be expressed as:

TD = [[Depart1, MNode], [UserA, user, ID, hash(UserA)], [Login D1, t]]

where, User A is an employee of “Depart1” (short for department) and department 1 is a “MNode” (short for management node); “UserA” is the name of user A, whose type is User, and if the type is user, then its user ID is data format. The hash value is hash (UserA). “Login D1” means logging in to the device 1. “t” is the time the user log in on device 1.

If it is another type of data, such as a file. User B creates a file named file1, then the structure can be represented as:

TD = [[UserB, PNode], [file1, file, do cx, hash(file1)], [Create on D2, t]]

where, “file1” belongs to the User B and “UserB” is a “PNode” (short for participating node); “file1” is the name of the file, whose type is file, and it is a word file so the format field is “docx”, and its hash value is hash(file1); “Create on D2” means user B create this file on device 2 and “t” is the time when user B create the file.

In this way, the system can adequately represent the state changes and their affiliation of all data objects in the internal network system, and the time when the state changes. Meaning, the data state changes could be traced according to the timestamp. However, this cannot guarantee the integrity of the data, because any user can delete, modify, or even forge non-existent traceability data. Therefore, storing these traceability data on the blockchain can not only ensure the reliability of the data but also ensure that the data will not be tampered with.

### 4.3. The Structure of Transaction and Block

This paper designed transaction and block based on traceability requirements for insider threats. Both transaction and block are actually a kind of data structure.

**Figure 4:**
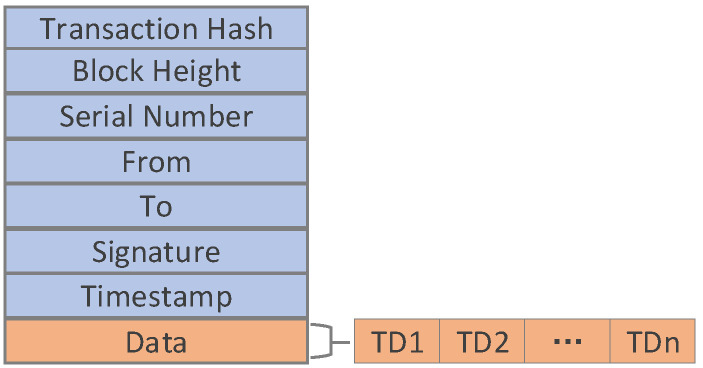
The structure of the transaction.

The role of the transaction is to package our TD data and identify the ID of the transaction (the hash value of the transaction). Figure 4 describes the structure of the transaction. The “Transaction Hash” stores the transaction ID. The “Block Height” is used to identify the height of the block where it is located. The “Serial Number” is used to mark the sequence in a block, and the “Timestamp” is used to mark the time when the transaction is sent. The “From” and “To” fields respectively indicate the address of the node that initiated the transaction and the address of the node that received the transaction. These addresses are usually represented by the hash of the node’s location information and should be unique in the system. The “Signature” field refers to the digital signature of the user who sent the transaction. The signature can be used to verify the authenticity of the transaction. The “Data” field is used to store traceability data structures, and each transaction may store multiple traceability data structures.

**Figure 5:**
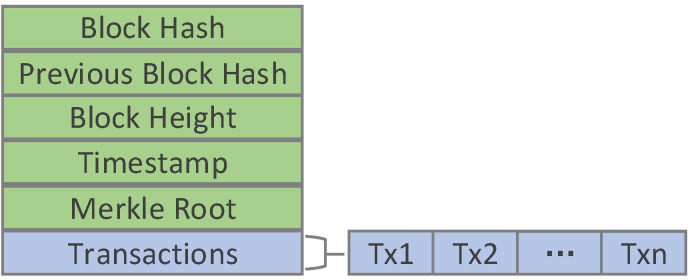
The structure of the block.

Similarly, the role of the block is to package transactions. As shown in Figure 5, the block of this system includes the block header (indicated by green) and block body (indicated by light purple). The block header includes: the “Block Hash” stores the block ID (the hash value of this block), the “Previous Block Hash” stores the previous block’s ID, the “Block Height” is used to mark the sequence of block is in the chain, the “Timestamp” stores the time when the block is generated, and the “Merkle Root” stores the Merkle root hash. The Merkle root hash ensures that any block in the blockchain network is modified will cause changes in Merkle root. The block body stores transactions and each block body may store multiple transactions. Therefore, each transaction has a unique serial number in a block.

**Figure 6:**
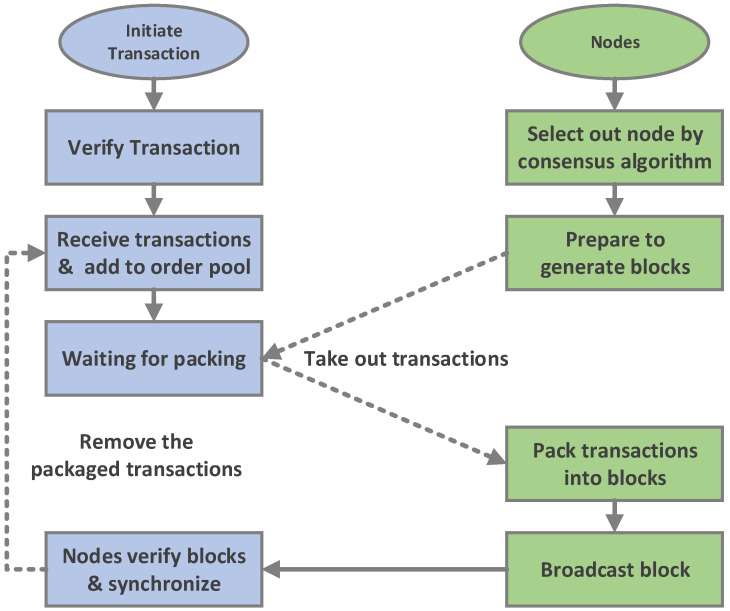
Flowchart of transactions from initiation to packaging to block.

The traceability data structure, transaction, and block all have their own timestamps. This is because the time to generate the data structure, the time to start the transaction and the time to generate the block may be different. All of them are recorded to make traceability more accurate. Figure 6 is the flowchart that depicts the process of transactions from initiation to packaging into a block.

### 4.4. Differential Privacy

In the blockchain system, any blockchain user or node can obtain data on the blockchain. This characteristic is also possible to be a potential threat to the network system. Because even within the organization, each department does not want the sensitive information of its own department to be obtained by other users. Therefore, technical measures are needed to protect some necessary sensitive information. However, considering that if the data on the blockchain can be used reasonably, it can bring potential value to the organization. For example, the large amount of available data is very important for the training of machine learning models. The traditional encryption method can protect privacy well, but it destroys the statistical characteristics of the data. Hence, this paper applies differential privacy technology to achieve the protection of sensitive information.

Specifically, this paper uses local differential privacy technology at each node. Make a random response before the user send the data TD, to get the actual send data Diffp(TD) with the privacy parameter ε. Thus, differentiated privacy protection can be achieved. The privacy parameter ε is a parameter that measures the degree of protection. The larger the ε, the smaller the protection. On the contrary, the smaller the ε, the higher the degree of data protection. The differential privacy algorithm is as Algorithm 1 shows. For example, assuming the data TD generated by participating nodes:

TD = [[DataOwnerName, DataOwnerTpye], [DataName, DataType, DataFormat, DataHash], [Activity, Timestamp]]

If the part that needs privacy protection is [DataType], [DataFormat], [DataHash], [Activity]. For each attribute (such as [Activity]), assuming it has N possible values, it may randomly return any one of the N values. Then the random response mechanism can be presented with the strategy matrix *Q*,

$$Q=\frac{1}{e^{\varepsilon }+N-1}\left[e^{\varepsilon }\underset{N-1}{\underbrace{1\cdots 1}}\right],$$

where $e^{\varepsilon }$ represents the probability of returning the correct value of the current attribute, and 1 indicates the probability of returning other attribute values. After getting the random response values of all the attributes in sequence, combine them and replace the original real values. At last, hashes TD and to get the Diffp(TD), then send Diffp(TD) to the management node. The final Diffp(TD) is formed as:

Diffp(TD) = [[DataOwnerName, DataOwnerTpye], [DataName, Diffp (DataType), Diffp (DataFormat), Diffp (DataHash)], [Diffp (Activity), Timestamp], hash(TD)]

**Algorithm 1** Algorithm for Differential Privacy.**Input:** data: TD, privacy budget: ϵ
**Output:** data with differential privacy protection: Diffp(TD)
1:**for** attribute $t_{i}$ in TD **do**2:** if** attribute $t_{i}$ needs privacy protection **then**3:  N = the number of candidates for attribute $t_{i}$4:  $t_{i}$ is real value with probability $\frac{e^{\epsilon }}{e^{\epsilon }+N-1}$5:  $t_{i}$ is other candidate value with probability $\frac{1}{e^{\epsilon }+N-1}$6:** end if**7:**end for**


### 4.5. Consensus Algorithm

The mainstream PoW (short for Proof of Work) algorithm currently used in Bitcoin and Ethereum is based on the principle that participants compete for the right to generate blocks by calculating the hash value of the block header. That is, the greater the amount of calculation and the more attempts, the greater the probability of obtaining the correct answer. This mechanism can attract more participants and increase the enthusiasm of participants, but it also causes unnecessary waste of resources. Obviously, the PoW algorithm is not suitable for an internal network system. Our system actually only serves the organization itself or members of the organization, so it is more reasonable to adopt the form of the private chain or the alliance chain. This paper consider the idea of using DPoS (short for Delegated Proof of Stake) consensus algorithm and make improvements based on the actual application.

The DPoS algorithm is characterized by a “witness node” concept, which can generate blocks. The generation of the witness node is elected by all shareholding nodes, and the top N candidates in the total number of consent votes can be elected as the witness node. This N value needs to be satisfied: at least half of the participating voters believe that N has been fully decentralized, and preferably an odd number.

Since the system is oriented to an internal network with high-security requirements and containing sensitive information, the structure of this network is relatively stable, and the ownership and authority of various departments in the network are clear. Therefore, this paper removes the DPoS voting process and directly use the nodes of various departments as witness nodes, called management nodes. Other nodes that only participate in generating traceability data are called participating nodes. Set a fixed time T, and randomly select a management node as a new block generation node at each T time. The system does not require cryptocurrency to allocate equity, so this is a cryptocurrency-free blockchain system. The advantage of this is that it can further reduce the energy consumption of the entire blockchain system, reduce network operating costs, and accelerate the consensus speed.

The basic idea of our consensus algorithm can be summarized as the following: every T time, all management nodes are randomly arranged. The first node is responsible for generating new blocks. Once the first node failed to complete the task, the second management node will generate the block, and then loops in turn, as Algorithm 2 shows.
**Algorithm 2** Algorithm for Consensus.**Input:** set of management node: D, the number of management node: N, fixed time: T, current time: t
**Output:** selected management node ID: SN
1:**if**$\left(\frac{t}{T}=0\right)$**then**2: Random(D) ← random arrangement D.3: D’ ← Random(D).4:** while** Select node sequentially from set D’ **do**5:  let SN ← D’.6:**  if** SN can generate blocks **then**7:**   return** SN8:**  else**9:   continue.10:**  end if**11:** end while**12: Let SN generate Block.13:**end if**


Each node in our blockchain system has two blockchains, a primary chain (privacy-chain) that all participated nodes maintain together and a secondary chain (self-chain) that each node maintains itself. That is, there is only one privacy-chain, and there are as many nodes as self-chain. Therefore, a primary and secondary chain synchronization algorithm is designed in this paper to synchronize the self-chain in each node with the privacy-chain block, as Algorithm 3 shows. Every T time, the management node packages all Tx within this period to generate a new block. This new block will be added to the privacy-chain, and all other nodes will also synchronize the new privacy-chain. When the node synchronizes to the privacy-chain when a new block is added, it will compare the Tx.Diffp(TD) in the new block with the TD it owns. If hash (TD) = Tx.hash (TD), make a copy of the new block, replace the corresponding Tx.Diffp(TD) with TD in the copy to form a new replica block, and add it to the node’s own self-chain.
**Algorithm 3** Algorithm for primary and secondary chain synchronization.**Require:** Transactions in txpool: Tx, fixed time: T
1:**while** Every T time **do**2: package all Tx in txpool into new block BLOCK ← Generate block.3: Add new BLOCK to the privacy-chain ← Update chain.4:** if** Nodes sync to privacy-chain with new BLOCK **then**5:  Each node will compare the Tx.Diffp(TD) in the new BLOCK with the TD they have.6:**  if** hash(TD) = Tx.hash(TD) **then**7:   Make a copy of the new BLOCK.8:   Replace(Tx.Diffp(TD), TD) ← Replace Tx.Diffp(TD) with TD to form a new Replica-Block.9:   add Replica-Block to the node’s own self-chain.10:**  end if**11:** end if**12:**end while**


The primary and secondary blockchains formed in this system have three characteristics: First, the traceability information in the privacy-chain only contains Diffp(TD), which means that sensitive information is protected by differential privacy, and no other node or department member can obtain the user’s privacy information from the privacy-chain; Second, the self-chain of the participation node only has the TD generated by the node’s own user, and the privacy information of other users can only be queried to Diffp(TD); Third, the self-chain of the management node has all the TD information of the participation nodes managed by the department, that is, the true source information TD of a user can only be queried in the self-chain of the user’s own participation node and the management node to which it belongs.

### 4.6. Traceability Data Storage and Query

#### 4.6.1. Traceability Data Storage Algorithm

The algorithm describes the process of generating TD data, performing differential privacy processing on TD data, forming transactions, and finally storing TD data on the chain. As Algorithm 4 describes, the basic process can be summarized as the following: When a user generates TD data, the participation node processes TD into Diffp(TD) through a differential privacy method, and hashes the TD, then get the TD’ = [Diffp(TD), hash(TD)] data. Users sign TD and TD’ separately, and then package TD’ to form the transaction Tx. A Transaction may contain multiple TDs. The participation nodes respective send Tx and TD to their management nodes. The management node hashes the TD and compares it with the hash (TD) in Tx. If they are consistent, put Tx and TD into txpool and tdpool respectively and wait for new block generation. Otherwise, it means that the TD may have been tampered with, and the management node will not add the corresponding Tx to the pool. At the same time, our blockchain system will also connect to other security systems (e.g., SOC to alarm).
**Algorithm 4** Algorithm for store traceability data.**Require:** traceability data: TD, fixed time: T
1:TD was generated on the participation node.2:Diffp(TD) ← Differential privacy processing of TD.3:[Diffp(TD), hash(TD)] ← Hash TD to form private-chain TD data.4:Sign(TD); [Sign(Diffp(TD)), hash(TD)] ← User signature.5:Tx = [Txhash, BH, SN, FROM, TO, NSign, TS, Sign(Diffp(TD)), hash(TD)] ← Generate transaction.6:**while** Every T time **do**7: send(Tx) to MNode ← PNode Send Tx to its MNode to which it belongs.8: send(TD) to MNode ← PNode Send TD to its MNode to which it belongs.9:** if** hash(TD) = Tx.hash(TD) **then**10:  Put Tx into txpool; Put TD into tdpool ← Put Tx and TD into the buffer pool and wait for block generation.11:** else**12:  continue.13:** end if**14:**end while**


#### 4.6.2. Traceability Query Algorithm

The algorithm describes how to trace objects from the blockchain system. As Algorithm 5 describes, the basic process can be summarized as the following: query the object obj in the privacy-chain, and the system returns all Diffp(TD) containing obj and the corresponding blockheight. Sort by timestamp to form a traceability information chain C1. Note that the TD information in C1 is the processed Diffp(TD), and does not contain real user behavior. Thus, according to Diffp(TD) query the management node it belongs to. The management node returns the corresponding TD according to blockheight and hash (TD). Finally, the real traceability information chain C0 of the object obj can be obtained.
**Algorithm 5** Algorithm for traceability query.**Input:** Query object: obj
**Output:** traceability information chain: C0 and C1
1:**while** All obj have been queried on the privacy-chain **do**2: (Diffp(TDn), BHn) ← Find the obj on the privacy-chain, BH is the height of the block.3: Add (Diffp(TDn), BHn) to the C1.4:**end while**5:Then get a complete C1 and Sort it by timestamp.6:C1 = (Diffp(TD0),BH0)←(Diffp(TD1),BH1)←...← (Diffp(TDn), BHn).7:**while** All obj have been queried in C1 **do**8: According to the (Diffp(TDn),BHn), Query data from its management node.9: The management node query TD from its self-chain based on BHn and its hash(TD).10: Add TDn to the C0.11:**end while**12:Then get a complete C0.13:C0 = TD0←TD1←...←TDn.


## 5. Experiment and Discussion

### 5.1. Experiment

**Figure 7:**
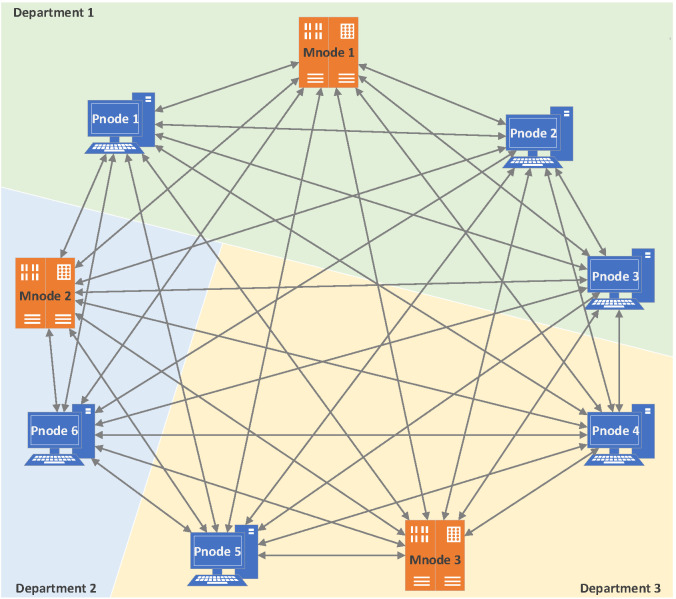
Topological diagram of the experimental environment.

According to the insider threat model and system architecture design, we have implemented the traceability blockchain system proposed in this paper and completed experiments on this basis. The traceability blockchain system is constructed in a simulated environment, including three different departments, three management nodes, and six participation nodes. The experimental topology is shown in Figure 7, where Mnode is the management node, Pnode is the participation node, and the department refers to the three departments to which they belong. In the experiment, differential privacy protection was only applied to [activity] (as mentioned in Section 4.2).

The purpose of our experiment is to verify the function of the system designed in Section 4 and to evaluate the performance of the system. To this end, we created four sets of tests:

1. Test the generation of TD data (as mentioned in Section 4.2), the Diffp(TD) processed by differential privacy (as described by Algorithm 2 in Section 4.4), and the process of packaging data into a transaction (as mentioned in Section 4.3). We record the time from TD generation to send to Mnode (hereafter referred to as T1).
2. Test the generation of a new block (as described by Figure 6 in Section 4.3). Notice that the processes of Test1 and Test2 are consistent as Algorithm 5 in Section 4.6.1. We record the system response time from Mnode packaged transaction to new block generation (hereafter referred to as T2).
3. Test the consensus mechanism of the system. After the new block is generated, the privacy-chain in all nodes will synchronize the new block through the consensus algorithm (as described by Algorithm 3 in Section 4.5), and each node will also update their self-chains (as described by Algorithm 4 in Section 4.5). We record the consensus response time, which is the system response time from the generation of new blocks to the final synchronization of privacy-chain and self-chain of all nodes (hereafter referred to as T3).
4. Test the query function of the system (as described by Algorithm 1 in Section 4.6.2). We record the time of traceability query, which is the system response time for a traceability object from query to return the traceability chain (hereafter referred to as T4).

**Figure 8:**
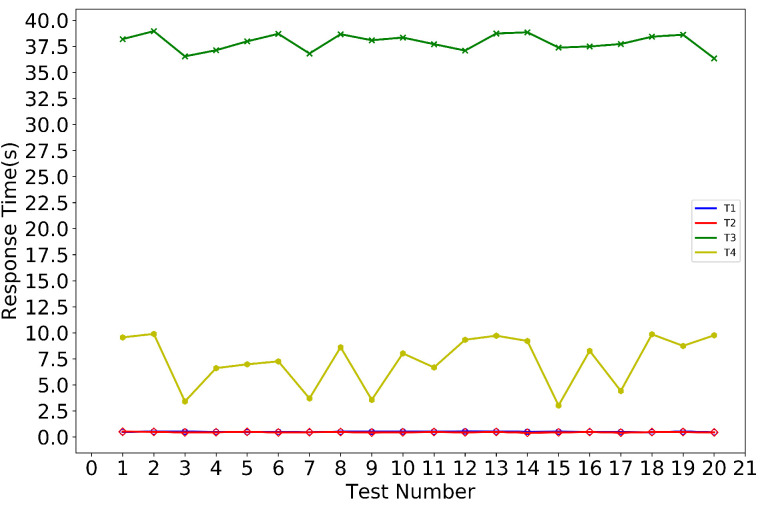
Results of system performance evaluation.

In total, the experiment was repeated 20 times and the results are shown in Figure 8. T1 takes between 451 ms and 523 ms, T2 takes 403 ms between and 498ms, T3 takes between 36.578 s and 38.794 s, and T4 takes between 2.698 s and 9.814 s. Except for T4, the other three spent a relatively constant time. This is related to querying the number of management nodes. When querying the traceability object, if the traceability result only needs to query one management node, it takes less time, and if it needs to query all the management nodes, then it takes more time.

**Figure 9:**
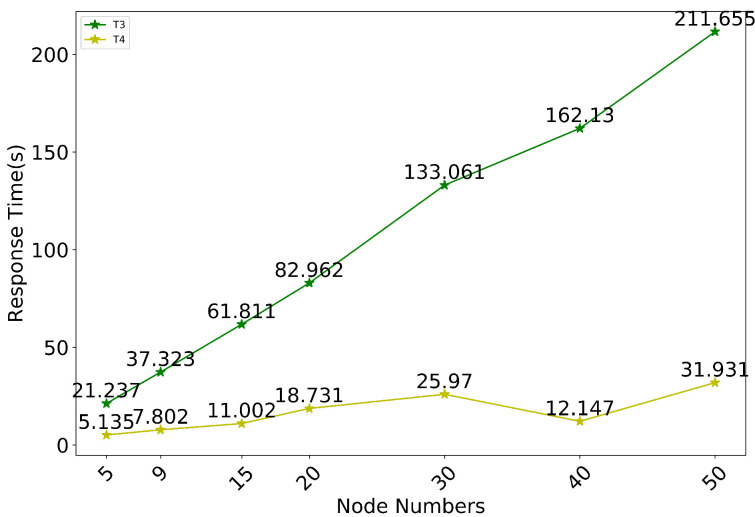
Evaluation results of the impact of nodes number on system performance.

The impact of additional nodes on system performance was evaluated by adjusting the number of nodes. The time spent by T1 and T2 relative to T3 was negligible and therefore was not counted. From Figure 9, it can be observed that as the number of nodes increases, the increase in the number of requests that need to be initiated at the time of consensus leads to a significant increase in T3 time. However, since the traceability query is related to the number of management nodes that need to be queried, T4 and node increase are not positively correlated.

### 5.2. Discussion

This paper builds an insider threat model of the internal network based on real insider threat situations. The blockchain system is also designed in terms of data structure, transaction structure, block structure, consensus algorithm, data storage algorithm, query algorithm, etc., and incorporates differential privacy to protect user data security. By developing this blockchain system and performing an experiment in a simulated environment, the results show that the blockchain can achieve the original intention of mitigating internal threats.

However, there are also some limitations. First, the experiment was performed in a small local area network. In a real network environment, performance may be reduced due to network latency. Second, the time interval for participation nodes to package traceability data, and the time interval for the management node to generate blocks are worthy of further research and testing. Inappropriate settings may cause the blockchain to fork. Third, the system can also become invalid in cases where there are too few nodes, such as when entire departments are malicious nodes that collectively tamper with records to send fake data. In summary, we will continue to address these issues, and in the future work, we will also conduct in-depth research on the security of blockchain technology itself.

## 6. Conclusions

In internal network systems, especially those containing sensitive information, the insider threat is one of its major security issues. At the same time, it is very difficult to track and obtain evidence of insider attacks. As a result, the attacker may launch multiple attacks and escape the tracing, which exacerbates the severity of the internal threat. This paper proposed a blockchain traceability system for insider threats to mitigate the issue of tracking attackers and obtaining evidence after the insider threat happened. We demonstrate the effectiveness of this blockchain traceability system by developing it and experimenting with it in a simulated environment to evaluate its performance. Our future work is to improve the privacy and performance of the proposed blockchain-based traceability system.

## Abbreviations

The following abbreviations are used in this manuscript:

ϵ	Differential privacy parameters
*Q*	Strategy matrix
IDS	Intrusion Detection System
IPS	Intrusion Prevention system
SOC	Security Operations Center
SIEM	Security Information and Event Management
Depart	Department
MNode	Management Node
PNode	Participating Node
PoW	Proof of Work
DPoS	Delegated Proof of Stak
